# Primary Colonic Diffuse Large B-cell Lymphoma: A Diagnostic Challenge

**DOI:** 10.7759/cureus.115132

**Published:** 2026-08-25

**Authors:** Samuel Montero

**Affiliations:** 1 Emergency Medicine, National Hospital, Panama, PAN; 2 Medical Education, Latin University of Panama, Panama, PAN; 3 Medical Internship, Complejo Hospitalario Dr. Arnulfo Arias Madrid, Panama, PAN

**Keywords:** cd20, colonic mass, diffuse large b cell lymphoma (dlbcl), inflammatory bowel disease, non-hodgins lymphoma, primary colonic lymphoma

## Abstract

Primary colonic diffuse large B-cell lymphoma (DLBCL) is a rare extranodal malignancy that accounts for a small percentage of colonic malignancies. With nonspecific gastrointestinal symptoms such as weight loss, abdominal pain, and bloody stools, it may mimic the presentation of inflammatory bowel disease. Symptoms usually present in advanced stages of the disease.

We present the case of a 19-year-old man with a history of one year of intermittent abdominal pain, unintentional weight loss, diarrhea, and bloody stools. Initial abdominal ultrasound reported distended bowel loops with no evidence of masses. During hospitalization, the patient underwent a colonoscopy, which revealed a transmural exophytic mass in the cecum near the ileocecal valve. A biopsy was taken, and findings suggested chronic inflammatory colitis. The patient was treated initially with corticosteroids and subsequently discharged with oral prednisone. However, four weeks later, the patient presented with persistent symptoms and recurrent episodes of bloody stools, which prompted admission and a second colonoscopy. During his admission, progressive intestinal obstruction prompted a right hemicolectomy.

Histopathological examination of the surgical specimen revealed DLBCL with CD20 positivity on immunohistochemistry, establishing the diagnosis of primary colonic lymphoma. This case highlights the diagnostic challenge posed by primary colonic DLBCL in young patients and emphasizes the importance of clinical suspicion and maintaining a broad differential diagnosis when histopathological findings are inconclusive.

## Introduction

Primary colorectal lymphomas are uncommon extranodal malignancies, representing less than 1% of all colorectal malignancies [1-4]. While diffuse large B-cell lymphoma (DLBCL) is the most common type of non-Hodgkin lymphoma (NHL), accounting for roughly 31% of all NHL in adults [4], the diagnosis of a primary colorectal lymphoma is exceedingly rare, with an estimated incidence of 0.2-0.6% among colonic malignancies [5]. Clinical manifestations are frequently nonspecific and include weight loss, nausea, vomiting, abdominal pain and distention, altered bowel habits, and bloody stools [2-7]. The cecum is among the most frequently involved colonic sites, possibly because of its abundant lymphoid tissue [1,4,6]. DLBCL is one of the most frequently encountered histologic subtypes of colorectal lymphomas. Primary colonic lymphomas have a predominance in male patients, with a mean age of 55 years and increasing incidence as age progresses [1,8,9].

This case warrants documentation as increased awareness of this rare diagnosis may help clinicians maintain a broad differential diagnosis, especially when evaluating colonic lesions in younger individuals. The findings in a primary colonic lymphoma frequently resemble those of chronic colitis or adenocarcinoma, further contributing to its diagnostic challenge. Clinical suspicion should prevail when the patient's signs and symptoms do not respond as expected to standard treatments or when histologic reports are inconclusive.

## Case presentation

In August 2022, a previously healthy 19-year-old male with a one-year history of intermittent abdominal pain and unintentional weight loss presented to the emergency department with severe abdominal pain, distention, and multiple episodes of bloody diarrhea. On admission, he was hemodynamically stable and afebrile. Physical examination revealed a distended abdomen with no rebound or tenderness. Laboratory evaluation revealed mild anemia without leukocytosis, while nutritional markers were within normal limits (Table 1). 

**Table 1:** Laboratory findings at hospital admission

Laboratory Test	Result	Reference Range
White blood cell count (WBC)	4.44 × 10³/mm³	4.00-10.00 × 10³/mm³
Hemoglobin	11.40 g/dL	13.0-17.0 g/dL
Absolute lymphocyte count	1.07 × 10³/mm³	1.00-4.00 × 10³/mm³
Lymphocytes (%)	24.2%	25-40%
Creatinine	0.96 mg/dL	0.72-1.18 mg/dL
Blood urea nitrogen (BUN)	14 mg/dL	8-20 mg/dL
Albumin	3.6 g/dL	3.5-5.2 g/dL
Total protein	6.1 g/dL	6.0-8.3 g/dL

Initial laboratory testing reported negative HIV serology. Abdominal ultrasound reported distended bowel loops without evidence of masses or a plastron process. A contrast abdominal and pelvic CT scan was performed and described wall thickening in the distal ileum and ileocecal valve, extending throughout 7 cm of the colonic wall, and multiple reactive lymph nodes. Based on the signs, symptoms, and radiologic findings, the differential diagnosis included inflammatory bowel disease, infectious colitis, and colorectal malignancy. Admitted to the gastroenterology department, the patient underwent a colonoscopy, which described a circumferential ulcerated exophytic lesion involving the cecum. Initial biopsies were obtained for histopathological evaluation. The first biopsy demonstrated severe chronic active colitis with ulceration and a dense mixed inflammatory infiltrate. No epithelial malignancy was identified; fungal organisms and cytomegalovirus were excluded. Because the inflammatory infiltrate obscured the lesion, colorectal carcinoma was considered unlikely in the sampled tissue. During hospitalization, the patient was initially managed with IV steroids, and his symptoms improved in the following days. Since the patient remained stable, he was discharged with oral prednisone daily and outpatient follow-up in the gastroenterology clinics.

**Figure 1:**
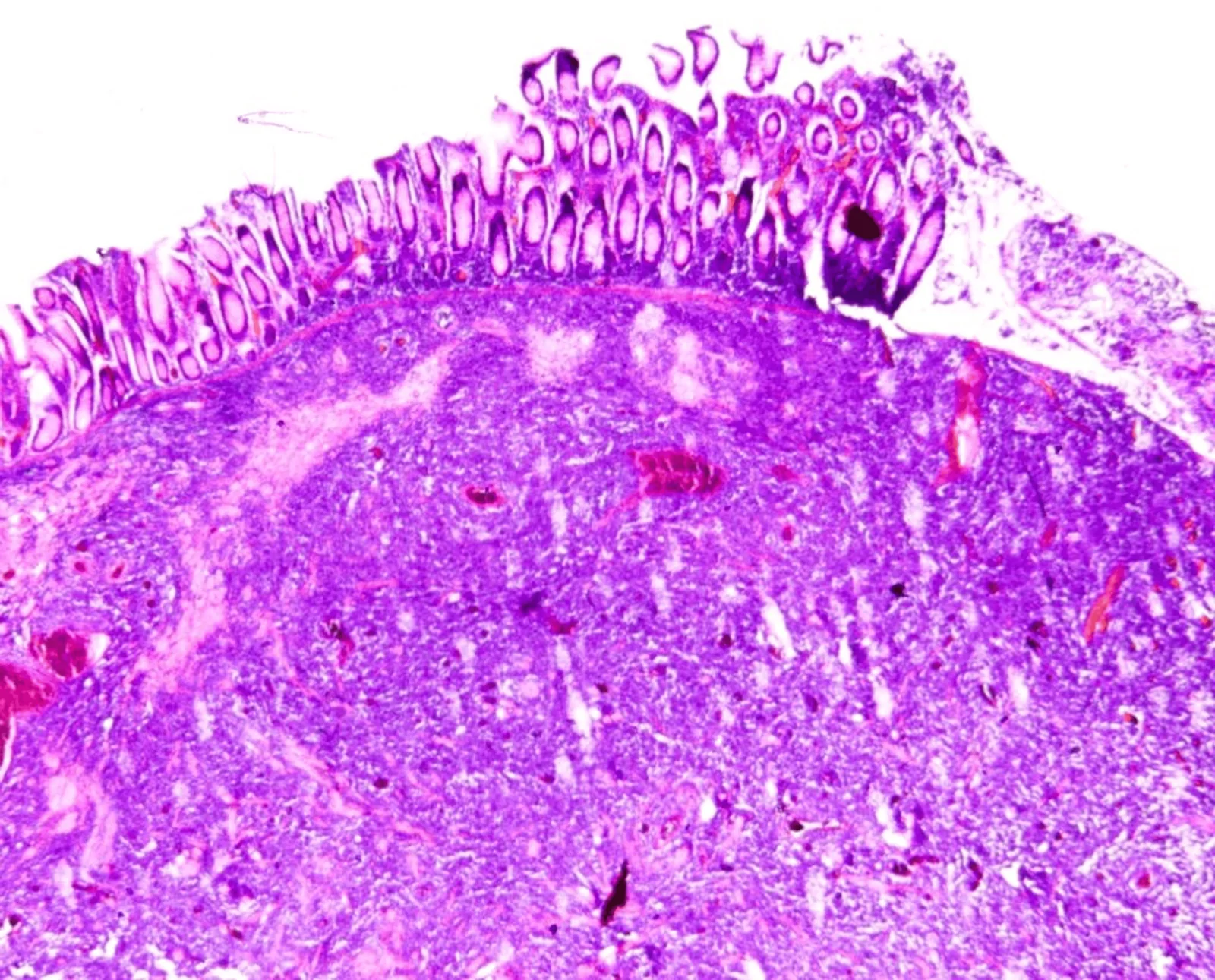
Low-power histology demonstrating preserved colonic mucosa, overlying a dense lymphoid infiltrate involving the bowel wall Hematoxylin and eosin stain (H&E), ×40.

In the following four weeks, the patient returned with a history of changes in his bowel habits, severe abdominal pain, worsening abdominal distention, and persistent episodes of bloody stools. Repeat colonoscopy revealed progression of the initial exophytic cecal lesion, which occupied approximately 70-80% of the intestinal lumen and was highly suspicious for malignancy. A second biopsy found ulceration associated with an atypical lymphoid proliferation. Immunohistochemistry of the specimen was negative for IRF4/MUM, with positivity for CD20 expression within the atypical cells and partial positivity for CD10, raising concern for B-cell lymphoma (Figure 1).

**Figure 2:**
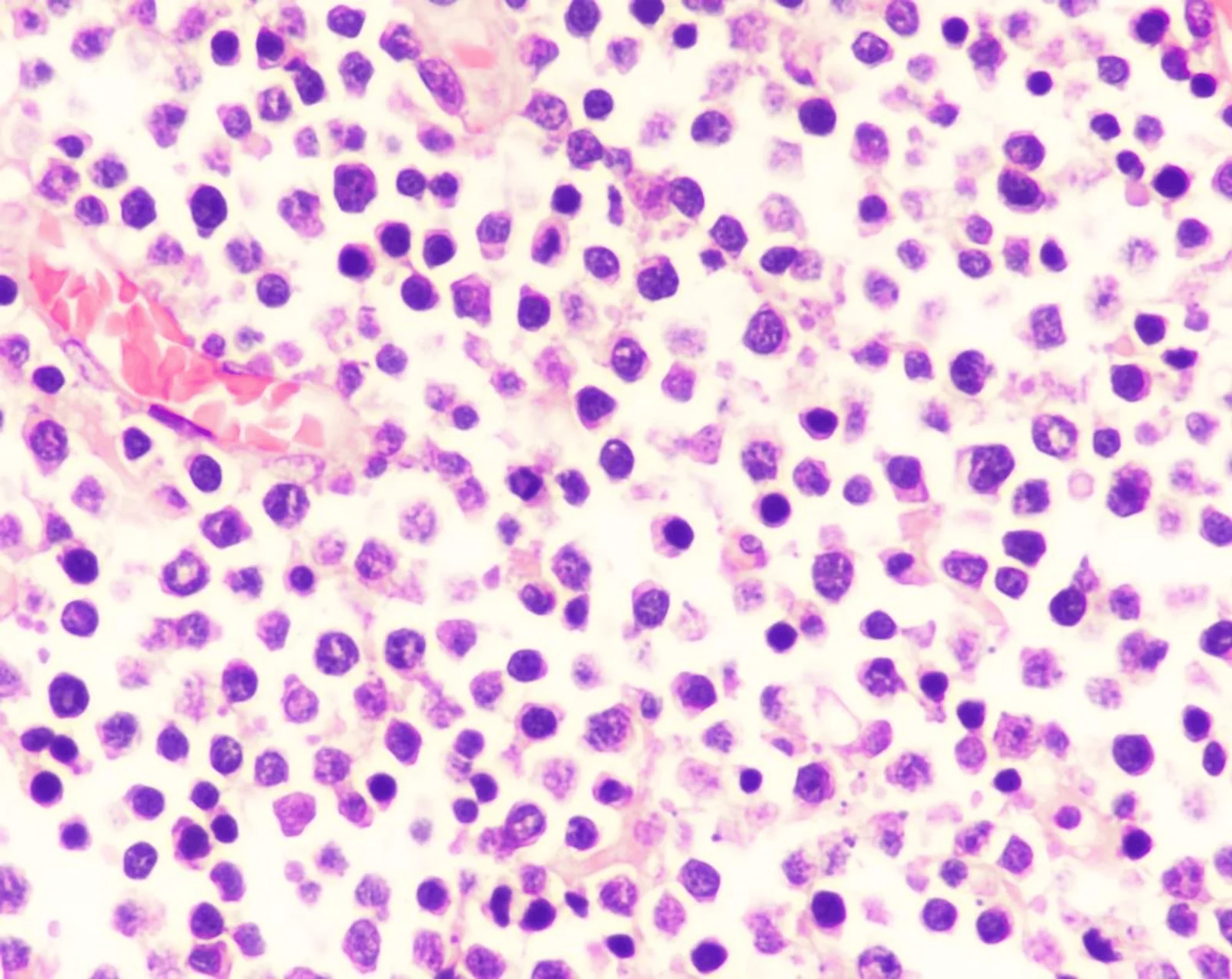
High-power section showing diffuse proliferation of atypical lymphoid cells with vesicular chromatin and prominent nucleoli. Immunohistochemical staining demonstrated CD20 positivity, supporting the diagnosis of a B-cell lymphoma Hematoxylin and eosin stain (H&E), ×400.

Due to progressive bowel obstruction and a high risk of perforation, the patient underwent right hemicolectomy with ileostomy. Gross examination of the surgical specimen showed a 9.0 × 5.5 cm polypoid lesion involving the ileocecal valve and cecum with complete luminal obstruction. Histopathological evaluation reported diffuse transmural infiltration by high-grade B-cell lymphoma composed of large atypical lymphoid cells with vesicular chromatin, prominent nucleoli, and frequent mitotic figures. Surgical margins were free of tumor (Figure 2). 

Immunohistochemical analysis was positive for CD45, CD79a, PAX5, heterogeneous CD20, and CD10, with focal BCL6 expression. The neoplastic cells were negative for BCL2, ALK, TdT, IRF4/MUM1, CD34, and CD138. EBER testing could not be performed because the reagent was unavailable. The Ki-67 proliferation index was approximately 80%, supporting the diagnosis of a high-grade DLBCL with a germinal center phenotype.

Postoperatively, the patient remained hemodynamically stable and reported improvement in his initial abdominal pain and distention. As part of the staging workup, a bone marrow biopsy demonstrated hyperplasia of the granulocytic and megakaryocytic lineages without morphologic evidence of lymphoma infiltration. Subsequently, the patient initiated treatment with the R-CHOP (rituximab, cyclophosphamide, doxorubicin hydrochloride (hydroxydaunorubicin), vincristine (Oncovin), and prednisone) chemotherapy regimen under the Hematology department; however, the patient was transferred to the Hematology service; therefore, his medical record, individual drug doses, long-term follow-up, and number of cycles were unavailable to the author.

## Discussion

Primary colorectal lymphoma is an uncommon malignancy, accounting for approximately 0.2%-1% of colonic malignancies in published series [1-6,8]. It predominantly affects elderly males, with the highest incidence generally reported during the sixth and seventh decades of life [2,4]. The markedly younger age of our patient therefore represents an unusual feature.

This case emphasizes that persistent clinical suspicion should prevail when endoscopic and histopathological results are inconclusive. Common colonoscopic findings range from strictures and ulcers to masses, intramural lesions, or diffuse polypoid lesions, which overlap the findings in adenocarcinomas and chronic colitis, further contributing to the diagnostic challenge [10]. While distinguishing between different diagnoses is particularly complicated during an endoscopic procedure, key findings have been described that might lead toward the definitive cause. Preserved overlying mucosa on a bulky submucosal lesion is a classic pattern produced by lymphomas. Ileocecal or terminal-ileal locations, where lymphomas have a predilection and primary adenocarcinomas are less likely, or ulcers within elevated lesions could hint toward a colonic lymphoma [1,11,12]. The use of narrow-band imaging (NBI) can help differentiate between adenocarcinomas and other types of lesions. With a real-time sensitivity of 91% and specificity of 83%, it is reliable for detecting primary neoplastic adenocarcinomas. For lymphomas, the sensitivity is roughly 63%, which may guide toward a lymphoproliferative cause but is not definitive for diagnosis [13].

Repeating biopsy, deeper tissue sampling, or surgical exploration may be required to establish the diagnosis in selected patients. The cecum and ileocecal region are commonly involved sites [2,6], which are important sites to explore when suspecting a colorectal lymphoma. Consequently, primary colonic lymphoma may initially resemble colorectal carcinoma, inflammatory bowel disease, or other inflammatory and infectious conditions. Therefore, close follow-up in the outpatient setting is key for detecting the disease and decreasing its complications.

Our patient illustrates the diagnostic difficulties associated with primary colonic lymphomas. Although the colonoscopic appearance suggested malignancy from the outset, the initial biopsy demonstrated only severe chronic active colitis. The extensive inflammatory infiltrate likely obscured the underlying lymphoma, delaying definitive diagnosis. Only after repeat tissue sampling and ultimately surgical resection was the diagnosis established.

## Conclusions

Primary colonic DLBCL should be considered in the differential diagnosis of young patients presenting with persistent gastrointestinal symptoms and suspected colonic neoplasms. Negative or nondiagnostic endoscopic biopsies do not exclude malignancy, particularly when clinical suspicion remains high. Early recognition and repeat tissue sampling may facilitate timely diagnosis and definitive treatment. The inflammatory process could delay diagnosis, but clinical suspicion is important, especially if the patient does not respond to conservative management.
